# Prolonging the Efficacy of Botulinum Toxin A Using a Novel Toxin Mimetic Complex and Peptide Formulation

**DOI:** 10.1111/jocd.71183

**Published:** 2026-09-17

**Authors:** Riad Kassem, Tomer Mimouni, Lee Magal, Ziad Khamaysi, Avner Shemer

**Affiliations:** ^1^ Department of Dermatology Sheba Medical Centre Ramat Gan Israel; ^2^ Gray School of Medical Sciences Tel Aviv University Tel Aviv Israel; ^3^ Shemer Clinic Netanya Israel; ^4^ Department of Dermatology Rambam Health Care Campus Haifa Israel; ^5^ Bruce Rappaport Faculty of Medicine Technion Institute of Technology Haifa Israel

**Keywords:** anti‐aging, botulinum toxin type A, cosmetic dermatology, dynamic wrinkles, neuromodulating peptides, Subderma‐Complex

## Abstract

**Background:**

Botulinum toxin type A (BTX‐A) is the cornerstone for treating dynamic wrinkles; however, it requires reinjection every 3–4 months. Adjunctive topical creams that mimic the effect of BTX‐A and enhance its action may prolong treatment intervals.

**Objectives:**

To evaluate the efficacy of a novel formulation containing Subderma‐Complex (arginine, niacinamide, and over‐the‐counter neuromodulating peptides, including: Munapsys, X50 Myocept, Syn‐Ake, and Argireline) in extending the duration of BTX‐A's effect.

**Methods & Materials:**

Subjects with moderate‐to‐severe facial wrinkles and a history of regular BTX‐A use applied the Subderma‐Complex serum and creams daily following BTX‐A injection. Reinjection was triggered by the reappearance of the first wrinkle. Outcomes were measured across five visits using the Investigator's Global Assessment, Subject Self‐Assessment, and instrumental analysis (VisioFace Lite).

**Results:**

Data from 15 participants were analyzed. The mean reinjection interval increased by 65.2%, from 13.5 ± 1.9 weeks at baseline to 22.3 ± 3.6 weeks following topical treatment (*p* < 0.001). Both investigator and patient assessments showed significant reductions in wrinkle severity and improvements in skin quality (*p* < 0.001). However, the small sample size and lack of a control group limit the generalizability of the findings.

**Conclusions:**

Daily application of a Subderma‐Complex formulation was well‐tolerated and significantly prolonged BTX‐A efficacy, potentially reducing reinjection frequency.

## 1 Introduction

Facial expression lines, resulting from repeated contractions of the underlying facial muscles, generate dynamic wrinkles that gradually progress to static wrinkles [1, 2]. With aging, the skin's resilience decreases owing to diminished collagen and elastin production, which reduces its capacity to repair continuous contraction‐induced microdamage [3]. This process is exacerbated by environmental factors, such as ultraviolet A and B exposure, that accelerate structural protein degradation [4]. To counteract these effects, strategies to reduce muscle activity through botulinum toxin injections and to enhance structural protein levels are used to preserve skin elasticity and appearance [5].

Botulinum toxin type A (BTX‐A), comprising onabotulinumtoxin A, abobotulinumtoxin A, and incobotulinumtoxin A, has remained a cornerstone in the treatment of dynamic wrinkles. The primary mechanism involves temporary paralysis of underlying muscle activity to provide smoother skin contours [6, 7, 8, 9, 10]. Within 1 week of application, approximately 80% of BTX‐A recipients achieve a noticeable reduction in wrinkle appearance; efficacy typically peaks at 1 month and gradually diminishes, lasting for 3–4 months [11, 12, 13]. BTX‐A's transient effect necessitates periodic reinjection to sustain cosmetic outcomes. Thus, interest has increased in adjunctive topical strategies to extend the antiwrinkle effects of BTX‐A and to simultaneously enhance skin health [14]. Innovations have focused on advanced peptide technologies that mimic the effects of the BTX‐A neurotransmitter at the neuromuscular junction, and these directly target the underlying causes of wrinkle formation by temporarily relaxing the superficial facial muscles. These peptides address aesthetic concerns while maintaining structural integrity, dermal support, and hydration [15, 16]. Peptide agents are incorporated in different formulations, including serums, creams, and facemasks. They offer a noninvasive alternative for wrinkle reduction and prevention. These over‐the‐counter (OTC) peptides offer advantages, such as ease of application, low incidence of adverse effects, and a budget‐friendly solution for maintaining naturalistic results [17, 18, 19, 20, 21, 22].

Peptides used in topical cosmetics include synthetic, animal‐derived, or plant‐based bioactive compounds that mimic BTX‐A's mechanism of action. The synthetic peptides include Argireline (Acetyl Hexapeptide‐8), Snap‐8 (Acetyl Octapeptide‐3), Leuphasyl (Pentapeptide‐18), and Vialox (Pentapeptide‐3). Animal‐derived peptides include: XEP‐30, XEP‐018 (μ‐conotoxin CnIIIC), and Syn‐Ake (dipeptide diaminobutyroyl benzylamide diacetate). Myoxinol, an okra derivative, is a plant‐based bioactive compound [17, 21].

This study aimed to evaluate the efficacy of novel creams and serum formulation containing Subderma‐Complex and neuromuscular modulating peptides in prolonging the BTX‐A‐injection‐induced clinical effect. A secondary objective was to evaluate changes in skin‐aging parameters and wrinkle severity during the study period.

## 2 Materials and Methods

### 2.1 Study Design and Participants

This investigator‐initiated, single‐center, clinical proof‐of‐concept study enrolled healthy male and female volunteers at Laniado Hospital in Israel. Eligible patients were those who presented with mild‐to‐moderate forehead, glabellar, and lateral canthal wrinkles at rest, and moderate to severe wrinkles during contraction. All participants received at least three consecutive BTX‐A treatments within the 12 months preceding study initiation and agreed to discontinue the use of wrinkle‐modifying cosmetic products.

Exclusion criteria included prior adverse reactions to BTX‐A, neuromuscular disorders, periocular surgeries, cosmetic hypersensitivity, and blood coagulation abnormalities. Written informed consent was obtained from all the participants. This study conformed to the principles of the Declaration of Helsinki and was approved by the Institutional Review Board of Laniado Hospital (IRB no. LND‐0026‐23).

The subjects were instructed to apply the serum and day and night creams following BTX‐A injection. The skin‐repair formulation contained Subderma‐Complex, which is a proprietary combination of arginine and niacinamide, alongside select OTC neuromuscular‐modulating peptides including: (1) Munapsys, which is a novel peptide complex that targets both pre‐ and post‐synaptic pathways to reduce muscle contractions and facial expression lines; (2) X50 Myocept, a smart‐delivery peptide system that selectively targets mimic muscles to modulate contraction and reduce dynamic wrinkles; (3) Syn‐Ake, a synthetic tripeptide that mimics the effect of Waglerin‐1 (a snake‐venom component) to block muscle receptors and smoothen expression lines [23]; (4) Argireline (acetyl hexapeptide‐8), a synthetic peptide that disrupts the protein complex which induces muscle signaling, inhibits the release of neurotransmitters that cause contractions, and effectively smooths expression lines. Furthermore, the creams contained niacinamide and hyaluronic acid to support the skin‐barrier function [17].

### 2.2 Outcome Measures

The primary outcome was the duration between injection and reinjection (weeks) compared to each participant's baseline expected interval. The secondary outcomes included the following evaluations on each visit:

1. Investigator Global Assessment (IGA) of wrinkle severity (0 = none; 1 = mild; 2 = moderate; 3 = severe).
2. Subject Self‐Assessment (SSA) of wrinkle severity.
3. Quantitative investigator analysis of the number and width of wrinkles > 1 mm and < 1 mm.
4. Instrumental image‐based wrinkle analysis using the VisioFace Lite system (Courage + Khazaka electronic GmbH, Germany) and Complete Skin Investigation (CSI) to measure the wrinkle volume (pixel^3^) and depth (pixel).
5. Investigator and subjective global assessment of skin quality and aging parameters
6. Instrumental evaluation of hydration and elasticity by Multi Skin Test Center MC1000 (Courage + Khazaka electronic GmbH, Germany).

### 2.3 Intervention and Study Visits

Five visits were conducted for each participant as follows: Visit 1: screening and assessment of wrinkle severity and skin aging; Visit 2: study initiation, wherein each participant received BTX‐A injections (Dysport, abbotulinum toxin A) and a vial of 500 units diluted in 2.5‐mL saline was injected according to the standard procedure for three regions, with 5 injection points for the forehead, three points for the glabella, and each lateral canthal region administering 5 units per point, using 30G needles, followed by daily application of subdermal post‐BTX‐A creams and serum, excluding the eyes; Visit 3: the first follow‐up visit 10–14 days after BTX‐A injection; Visit 4: the second follow‐up visit was timed according to each participant's known interval for regular BTX‐A reinjection; and Visit 5: the third follow‐up, occurring upon the return of the first wrinkle that typically marks the beginning of deterioration and the time for actual reinjection. The injection interval between Visit 2 and Visit 5 was recorded and compared with the expected baseline interval (the interval between Visit 2 and Visit 4).

### 2.4 Statistical Analysis

Data were analyzed in Excel and verified via double data entry and a Proc Compare Discrepancy Report to ensure accuracy. Statistical analyses were performed using SAS (SAS Institute Inc., Cary, NC, USA) and Microsoft Excel. Categorical and continuous variables are summarized using descriptive statistics. For the primary and secondary endpoints, including IGA scores, SSA scores, quantitative wrinkle counts, and instrumental volumetric/depth analysis (VisioFace Lite), the Wilcoxon Signed‐Rank test was used to compare changes from baseline to follow‐up visits. Statistical significance was set at *p* < 0.05.

## 3 Results

### 3.1 Patient Characteristics

Sixteen participants (15 women, 1 man; mean age, 44.6 ± 7.3 years; Table 1) were enrolled; however, one participant discontinued treatment owing to localized eyelid contact dermatitis caused by incorrect topical use, and 15 participants completed the study.

**TABLE 1:** Baseline characteristics of study participants (*n* = 15).

Characteristics total (*n* = 15)	
Sex, female, *n* (%)	14 (93.3)
Age, years, mean ± SD	44.6 ± 7.3
Fitzpatrick skin type, *n* (%)	
2	1 (6.7)
3	5 (33.3)
4	5 (33.3)
5	4 (26.7)

### 3.2 Prolongation of BTX‐A Interval

**FIGURE 1:**
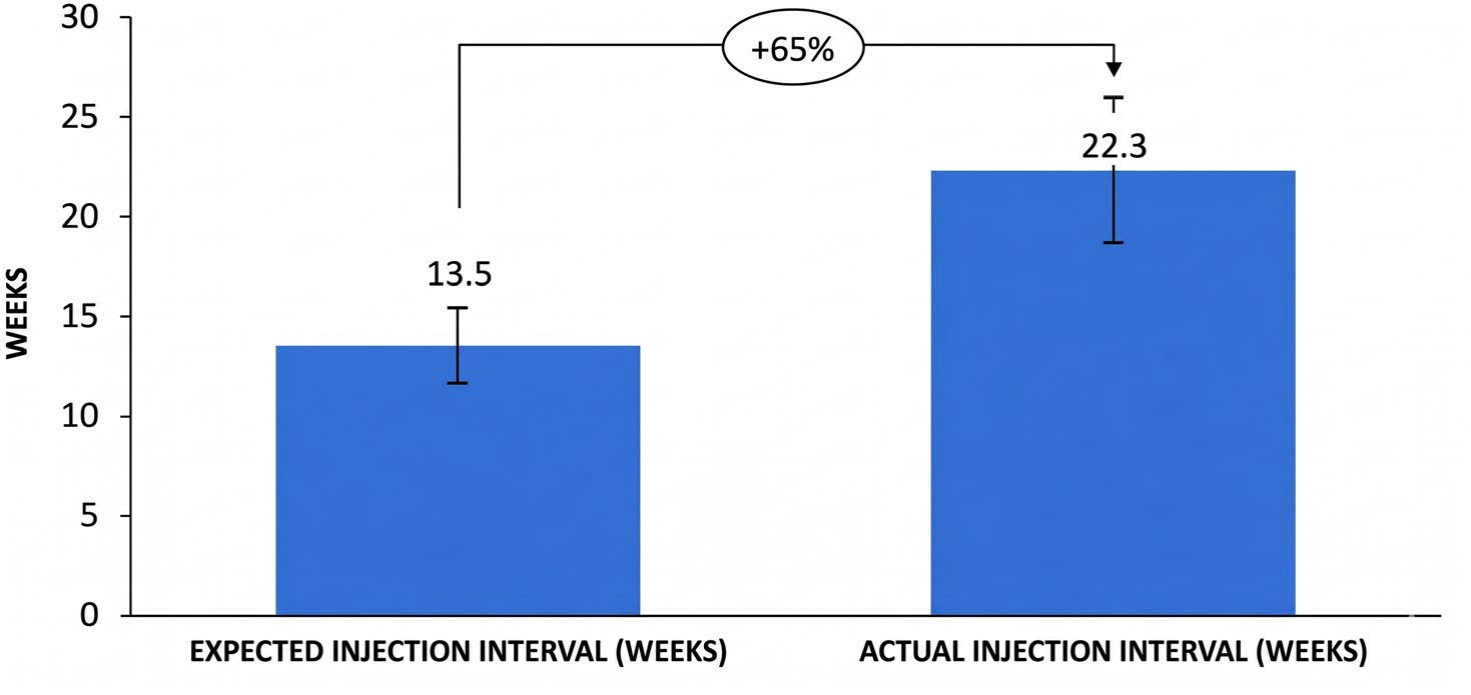
The mean expected injection interval (Visit 1 to Visit 4) vs. the mean actual injection interval (Visit 1 to Visit 5).

The subdermal post‐BTX regimen significantly extended the effects of BTX‐A injection treatment. Participants demonstrated a mean 65.2% increase from their expected historical injection interval to their actual injection interval (13.5 ± 1.9 weeks vs. 22.3 ± 3.6 weeks; *p* < 0.001; Figure 1).

### 3.3 Clinical and Instrumental Outcomes

The IGA scores showed significant and consistent improvements in dynamic and static wrinkle severity across all treated areas throughout the follow‐up visits (Table 2). Quantitative assessments confirmed significant reductions in both fine and deep wrinkles (> 1 and < 1 mm) at every follow‐up visit, whereas the non‐injected areas showed improved skin conditions. The SSA scores mirrored these findings, particularly with significant improvements in dynamic wrinkles throughout the follow‐up period (Table 2). Finally, instrumental analysis using VisioFace Lite and quantitative camera data corroborated these results and demonstrated progressive, substantial decreases in wrinkle volume and depth (*p* < 0.001 across multiple sites; Table 2).

**TABLE 2:** Investigator's Global Assessment (IGA) of wrinkle severity, Subject Self‐Assessment (SSA) of wrinkle severity by the participants, and wrinkle‐depth assessment by instrumental Complete Skin Investigation (CSI) analysis (VisioFace Lite) of the participants (*n* = 15). The following table shows the results of each assessment method in each location (static and dynamic) during baseline and visit, and the reinjection time for each participant.

Location	Assessment method^a^	Baseline, mean ± SD	Visit 5: reinjection time	*p*
Horizontal forehead, dynamic	IGA (0–3)	2.5 ± 0.6	1.5 ± 0.5	< 0.001
	SSA (0–3)	2.2 ± 0.6	1.5 ± 0.7	0.008
	CSI (Depth, pixel)	12.5 ± 2.9	8.8 ± 1.1	< 0.001
Horizontal forehead, static	IGA (0–3)	1.5 ± 0.7	0.4 ± 0.5	< 0.001
	SSA (0–3)	1.3 ± 1	0.8 ± 0.8	0.11
	CSI (Depth, pixel)	11.1 ± 2.5	8.1 ± 0.9	< 0.001
Glabella, dynamic	IGA (0–3)	2.4 ± 0.7	1.5 ± 0.6	< 0.001
	SSA (0–3)	2.2 ± 0.6	1.4 ± 0.7	0.009
	CSI (Depth, pixel)	16.3 ± 3.3	9.9 ± 1.8	< 0.001
Glabella, static	IGA (0–3)	1.3 ± 0.9	0.5 ± 0.5	< 0.001
	SSA (0–3)	1.2 ± 1	0.9 ± 0.8	0.43
	CSI (Depth, pixel)	15.7 ± 4.3	9.1 ± 1.3	< 0.001
Lateral canthal, dynamic	IGA (0–3)	2.7 ± 0.6	1.5 ± 0.7	< 0.001
	SSA (0–3)	2.5 ± 0.5	1.5 ± 0.6	< 0.001
	CSI (Depth, pixel)	17.8 ± 4.6	10.3 ± 1.9	< 0.001
Lateral canthal, static	IGA (0–3)	1.2 ± 0.9	0.3 ± 0.5	< 0.001
	SSA (0–3)	1.4 ± 0.7	0.7 ± 0.8	0.01
	CSI (Depth, pixel)	14.1 ± 3.4	9.3 ± 1.6	< 0.001

^a^ IGA and SSA scores: 0 = none, 1 = mild, 2 = moderate, and 3 = severe.

### 3.4 Adverse Events

One participant developed contact dermatitis of the eyelids owing to cream misapplication, and the symptoms resolved spontaneously after discontinuation. No local or systemic adverse reactions were observed.

## 4 Discussion

Psychological perceptions of aging, especially regarding the presence of dynamic wrinkles, significantly affect individuals, and many seek treatments to mitigate the visible signs of aging. Although BTX‐A remains the gold standard for temporary muscle paralysis, maintaining its benefits over longer intervals is highly desirable to enhance convenience, satisfaction, and cost‐effectiveness. By addressing these challenges and leveraging innovative technologies and regulatory improvements, the future of treating dynamic wrinkles can shift toward more effective, sustainable, accessible, and noninvasive options.

Topical botulinum toxin application can induce up to 25% improvement in wrinkle appearance after 4–8 weeks of daily application. Most topical botulinum‐toxin formulations are in the experimental or early commercial stages and require medical practitioner application, which is impractical in daily life [24, 25]. Although topical BTX‐A mimetic peptides cannot penetrate deeply enough to impact muscle contraction similarly to intramuscular injections, their influence on superficial facial tension and micro‐expression modulation supports prolonged aesthetic outcomes [26, 27, 28].

This study's findings suggest that incorporating topical “Botox‐mimicking” peptides can meaningfully extend the visible effects of BTX‐A injections by an average of 65.2%. Multiple assessment methods throughout the study showed significant improvements in wrinkle severity and other skin‐aging parameters, which reinforce the quality of the effect of the treatment. Patient satisfaction was notably high, with most participants appreciating the smoother transition between BTX‐A cycles and a decreased perception of the treatment “wearing off.” This contributed to a more continuous experience of aesthetic effects and general improvements in skin texture. The observed extension of the BTX‐A treatment interval offers numerous benefits to patients. Beyond the convenience of fewer clinic visits, this extension provides patients with a financial advantage by decreasing the total annual expenditure on BTX‐A treatment. These were consistent with the aesthetic outcomes. Combined with the enhanced skin texture, this outcome reinforced long‐term patient satisfaction and highlighted the strong safety profile of these agents when used appropriately.

Subderma‐Complex, which is formulated with argin‐ine, niacinamide, argireline, Munapsys, X50 Myocept, and Syn‐Ake, was designed to support post‐BTX skin recovery and enhance neuromodulatory activity. Interestingly, the combination of several topical agents yields a pronounced effect, which suggests a potential synergistic benefit when multiple peptides target different aspects of the neuromuscular signaling cascade. This additive mechanism is likely multifactorial, and includes a mild continuation of neuromodulatory effects at the superficial muscular level, strengthened skin‐barrier repair, and an enhanced local environment for neuromuscular recovery. Ultimately, the high satisfaction rates and extended injection cycles underscored the clinical practicality of this adjunctive protocol.

### 4.1 Limitations

The small sample size (*n* = 15) and absence of a placebo control group limit the generalizability of these findings. The reliance on subjective evaluation methods may have introduced bias. Topical absorption variability, patient adherence, and individual skin differences can influence outcomes. Furthermore, no biochemical or imaging confirmation (e.g., electromyography) was conducted to directly assess penetration and neuromuscular activity. Finally, the absence of a control arm to test the formulation without the proprietary complex prevented the isolation of the complex's specific contribution to the observed effect.

## 5 Conclusions

This proof‐of‐concept study provides preliminary evidence that a daily application of a topical formulation containing Subderma‐Complex can safely extend the clinical duration of BTX‐A efficacy. The results highlight a promising adjunctive noninvasive approach to reduce BTX‐A injection frequency and enhance patient satisfaction. Despite its limitations, this study highlights the potential of noninvasive adjuncts to provide a less aggressive approach to muscle relaxation. These findings support the use of multitasking topicals to bridge the gap between sessions, and offer a clinical alternative for patients who seek to minimize their total annual injections. Future large‐scale controlled studies with more objective metrics are warranted to validate these findings and establish standardized protocols for topical agent selection, concentration, application timing relative to BTX‐A administration, and long‐term safety. Future approaches to address dynamic wrinkles noninvasively involve enhancing penetration technologies, such as microencapsulation, nanotechnology, and skin‐permeation enhancers, to improve the delivery of active ingredients to deeper skin layers.

## Author Contributions

Conceptualization: R.K., A.S., L.M.; Data curation: R.K.; Methodology: R.K., A.S., L.M.; Formal analysis: R.K., A.S., L.M.; Writing – original draft preparation: R.K., T.M.; Writing – review and editing: R.K., Z.K., T.M.; Supervision: R.K.

## Funding

This investigator‐initiated study was conducted independently at the Research Center of Laniado Hospital, Netanya. The Subderma‐Complex products and BTX‐A (Dysport) utilized in this research were supplied by Lileger Ltd. (Herzliya, Israel). Lileger Ltd. provided no financial compensation, analytical support, or input regarding the study design, data interpretation, or manuscript preparation.

## Ethics Statement

The study protocol was approved by the Laniado Hospital Institutional Review Board (IRB No. LND‐0026‐23). All participants provided written informed consent prior to participating and publication of their data and any identifiable images. All procedures were conducted in strict accordance with the ethical principles outlined in the Declaration of Helsinki.

## Conflicts of Interest

The authors declare no conflicts of interest.

## Data Availability

The data that support the findings of this study are available from the corresponding author upon reasonable request.
